# Near-UVA Radiation at 405 nm: Germicidal Effect With and Without Sub-Lethal Conditions on *Gram*-Positive and *Gram*-Negative Bacteria

**DOI:** 10.3390/pathogens15010059

**Published:** 2026-01-07

**Authors:** Davide Amodeo, Giulio Pedrazzoli, Isa De Palma, Alessandro Puccio, Giacomo Trillocco, Gaia Papale, Gabriele Cevenini, Marco Tani, Gabriele Messina

**Affiliations:** 1 Department of Medical Biotechnologies, University of Siena, 53100 Siena, Italy; isa.depalma@student.unisi.it (I.D.P.); alessandropuccio@hotmail.com (A.P.); g.trillocco@student.unisi.it (G.T.); gabriele.cevenini@dbm.unisi.it (G.C.); 2 Department of Molecular and Developmental Medicine, University of Siena, 53100 Siena, Italy; g.pedrazzoli@student.unisi.it (G.P.); gaia.papale2@unisi.it (G.P.); gabriele.messina@unisi.it (G.M.); 3 Department of Information Engineering and Mathematics, University of Siena, 53100 Siena, Italy; tani@diism.unisi.it

**Keywords:** Near-UVA, 405 nm, disinfection, HAIs, bacterial inactivation

## Abstract

The need for contrasting Healthcare-Associated Infections requires the promotion and support of alternative disinfection techniques. Due to the antimicrobial potential of UV, devices equipped with UVC, UVB and UVA lamps or LEDs have been developed in recent years for domestic, everyday use. In this study, four bacterial strains (*S. aureus*, *E. faecalis*, *E. coli*, and *P. aeruginosa*) were exposed to different doses of near-UVA radiation at 405 nm, with an average irradiance of 21 mW/cm^2^, using an experimental multi-LED device. Bacterial suspensions were irradiated under both sub-lethal and non-sub-lethal stress conditions. When using only near-UVA light, the highest abatement effect was observed on *P. aeruginosa* (2.4 log_10_). Treatment with osmotic stress, in combination with light irradiation, was effective on all bacterial strains (mean abatement of 2.76, 5.46, 5.31, and 1.5 log_10_ on *E. coli*, *E. faecalis*, *P. aeruginosa*, and *S. aureus*, respectively). In heat stress conditions at 4 °C, *P. aeruginosa* and *S. aureus* species were the most susceptible (2.76 and 5.5 log_10_), whereas at 45 °C all species, except *E. faecalis* (0.58 log_10_), achieved significant reduction. The addition of exogenous photosensitive porphyrins produced a reduction in total concentrations from the lowest doses for *S. aureus* and *P. aeruginosa*, while for *E. coli* and *E. faecalis*, the reductions did not exceed 1 log_10_ abatement. Near-UVA radiation at 405 nm has a high disinfectant potential when combined with certain sub-lethal stress conditions. The most significant germicidal effect was achieved with the use of exogenous porphyrins in *S. aureus* and *P. aeruginosa* species. This study opens perspectives on the possible future application of near-UVA radiation in disinfection in order to limit the spread of healthcare-related infections.

## 1. Introduction

Environmental hygiene is crucial in healthcare settings to prevent Healthcare-Associated Infections (HCAIs).

*Escherichia coli*, *Staphylococcus aureus*, *Klebsiella pneumoniae*, *Streptococcus pneumoniae*, *Acinetobacter baumannii*, and *Pseudomonas aeruginosa* were the cause of at least 250,000 deaths each in 2019 [1]. This, in synergy with the recent SARS-CoV-2 pandemic, has contributed to an increase in collective interest in being able to limit the spread of difficult-to-treat infections, particularly in the context of healthcare-associated infections (HAIs) [2].

By definition, the main objective of disinfection is the reduction in the number of pathogenic micro-organisms to as close to zero as possible [3].

Evidence-based recommendations for the treatment of medical devices and healthcare environments define specific chemical disinfectants and sterilisation methods. The most commonly used chemical disinfectants include alcohols, glutaraldehyde, formaldehyde, hydrogen peroxide, iodophors, ortho-phthalaldehyde, peracetic acid, phenols, quaternary ammonium compounds and chlorine [3,4].

Recommended sterilisation methods include steam sterilisation, ethylene oxide (ETO), hydrogen peroxide gas plasma, peracetic acid, performic acid, ionising radiation, dry heat sterilisers, filtration, microwave, ozone, formaldehyde vapour and infrared radiation. When used properly, these processes can reduce the risk of infection associated with the use of invasive and non-invasive medical and surgical devices [3,5].

One physical disinfection technique that has seen a substantial increase, especially considering the recent pandemic, is the use of UV-C light. Although the methods and details of disinfection with ultraviolet light are fairly well understood, to the point that effective disinfection systems can be designed and installed with predictable effects, the exact nature of the effect of ultraviolet light on microorganisms at the molecular level is still a matter of intensive research [6]. In the health sector, UV lamps can be used to reduce the microbial load on the surface of surgical instruments, patient rooms and biological laboratories, where they are used daily to decontaminate the inside of hoods or metal and/or plastic objects [7]. Environmental sanitisation with chemical solvents combined with the use of UV technology has been shown to achieve deeper levels of disinfection and prevent HAIs [8].

The spectrum of ultraviolet light extends from wavelengths of about 100–400 nm. The subdivisions include UVA (400–315 nm), UVB (315–280 nm) and UVC (280–100 nm) [6].

In everyday practice in laboratories, operating theatres or ambulances, UV light sources are usually low-pressure mercury vapour lamps, which traditionally emit more than 90% of their spectral density at a wavelength of around 254 nm (UVC) [9]. This frequency is strongly absorbed by the nitrogenous bases in DNA and RNA. The aberrations caused by this energy transfer induce defects in DNA replication, resulting in a high germicidal potential [10].

However, this technology has limitations, as it cannot be used in the presence of people, making it unsuitable for continuous disinfection. Long-term use of UVC and UVB without appropriate precautions, as well as prolonged accidental exposure, could lead to the development of keratoconjunctivitis, basal cell carcinoma of the eye [11], melanoma, and other skin cancers among the possible associated diseases. UVA radiation can also induce melanoma formation by increasing oxidative stress levels in the melanocyte. This is because, although it is a less genotoxic wavelength than UVC and UVB, it has a greater penetrating capacity [12].

Recently, other light frequencies have been under study, driven by technological advancements allowing tailored ranges of wavelengths and the search for viable, sustainable, safe, and eco-friendly alternatives. N-UVA light, slightly above 400 nm, centred around the 405 nm wavelength, appears to be a potential option for continuous and safe disinfection systems.

These frequencies have germicidal effects based on the specific absorption of certain photosensitive molecules that contain tetrapyrrole rings in their structure, such as the porphyrin class, chlorins and bacteriochlorins [13]. These molecules interact with molecular oxygen to form Reactive Oxygen Species (ROS), which damage Gram-negative, Gram-positive bacterial and fungal species [14]. When comparing near-UVA light with UVC to achieve microbial inactivation, the former requires higher energy doses compared to the use of shorter wavelengths and therefore higher energy potential (UVC). However, near-UVA light has non-negligible advantages: (i) it is not harmful to humans at certain irradiation levels; (ii) it is less damaging to plastics (thus compatible with continuous use); and (iii) it can penetrate transparent surfaces such as plastics, glass and liquids [15].

Although there is already evidence on the disinfection performance of n-UVA light in controlled and real-world contexts [16,17], further investigations into the synergistic effects this light frequency may have with other study parameters on microbial species could be an interesting exploration to understand currently lesser-known mechanisms for optimizing the disinfection processes that this specific wavelength of light can provide. While the individual effects of 405 nm light have been explored, there is a lack of comprehensive data on how this radiation interacts with common environmental stressors. This study adopts a combined disinfection approach, investigating the synergy between near-UVA light and osmotic/thermal stress, providing new insights into optimizing disinfection protocols for complex real-world scenarios.

The aim of this study was to evaluate the germicidal effect of near-UVA radiation, at a wavelength of 405 nm, on the bacterial species *S. aureus*, *Enterococcus faecalis*, *E. coli* and *P. aeruginosa* at room temperature (22 °C and 36% humidity), heat stress (4 °C and 45 °C), osmotic stress (in 15% NaCI solution) and oxidative stress (using an exogenous porphyrin). These particular bacteria were selected because of their typical association with a wide range of infections in both nosocomial and community settings [18,19].

## 2. Materials and Methods

The experimental comparison study was conducted between November 2022 and January 2023 at the Environmental Hygiene Laboratory of the Department of Molecular and Developmental Medicine, University of Siena, Siena, Italy. The experiments were performed using a multi-LED (Light Emitting Diode) device capable of emitting near-UVA light radiation at a wavelength of 405 nm.

### 2.1. Setting

The experimental setup to determine the number and position of the LEDs in the device used to perform the microbiological tests was designed using Ansys Speos software ver. 2025 R2 (Ansys Inc., Canonsburg, PA, USA). Photometric simulations were performed to ensure good homogeneity of irradiance in all wells of the multi-well plate and to measure the spatial distribution of light irradiance at 405 nm.

**Figure 1:**
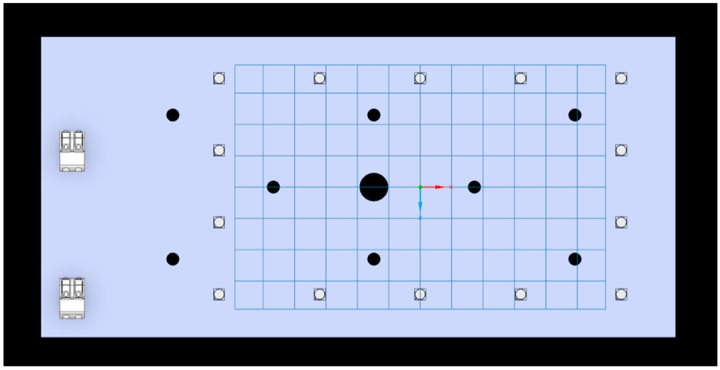
Top view of the design of the printed circuit board used for the 405 nm light experiments. The array of 14 LEDs (white dot in the picture) was arranged to illuminate the multi-well plate uniformly (from bottom to top) and positioned at a distance of approximately 10 cm.

The light source used consists of an array of 14 LEDs (Luminus SST-10-UV, Luminus, Brussels, Belgium) mounted on a printed circuit board (Figure 1).

**Figure 2:**
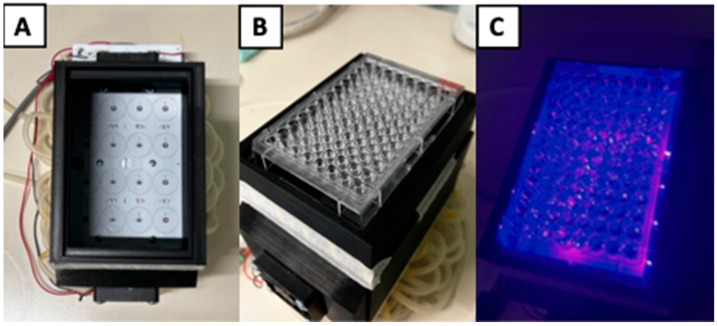
Multi-LED light source and PLA support used for the experiments. (**A**) On the left is a top view of the switched-off light source with the LEDs arranged in a grid and mounted on a PCB holder; (**B**) In the centre is the multi-well plate mounted on the PLA holder; (**C**) On the right, the n-UVA source is in operation.

**Figure 3:**
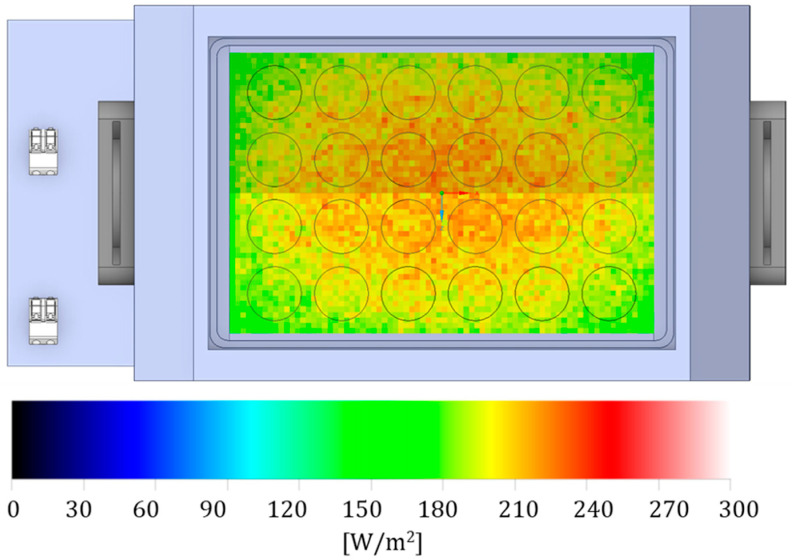
Radiometric simulation results in [W/m^2^] using Ansys Speos software. The image shows the light distribution in the different wells of the multi-well plate. Light scattering has been optimized to ensure an energy difference of approximately 10% between the outermost and central wells.

At the base, the source was connected to an aluminium heat sink that prevents the LEDs from excessively overheating and deteriorating, and avoids excessive heating of bacterial inocula, which could affect the experiment results. Above the light source, a rectangular black polylactic acid (PLA) stand (17 × 10 × 12 cm) was mounted to support the multiwell plate and to channel the light radiation (Figure 2). It was designed with CAD software Solidworks 2020 Professional (Dassault Systèmes, Waltham, MA, USA) and fabricated with a 3D printer (Ultimaker B.V., Utrecht, The Netherlands). Characterization of the electromagnetic spectrum was performed with the use of an Avantes ULS2048CL-EVO spectrophotometer (Avantes, Apeldoorn, The Netherlands). The LEDs provide unpolarized radiation with a peak wavelength of 405 nm and a narrow spectral bandwidth (Full Width at Half Maximum, FWHM) of 10 nm. The irradiance was maintained at a constant 21 mW/cm^2^ at the multiwell plate height. The multiwell plates used were optical-grade polystyrene, a material with a transmittance exceeding 90% at 405 nm, ensuring minimal loss of photon flux. Irradiation was performed from the bottom (below the wells) at a fixed distance of 10 cm, optimized to ensure radiometric homogeneity across the plate surface, as confirmed by Ansys Speos simulations (Figure 3). A measurement-based “energy map” of the multiwell plate was made in Excel to select the wells with the highest irradiance values, where the maximum energy difference between wells was 10% (Table 1).

### 2.2. Microbiological Procedure

Microbiological experiments were conducted on four bacterial strains: *E. coli* (ATCC 8739), *S. aureus* (ATCC 43300), *E. faecalis* (ATCC 51299), and *P. aeruginosa* (ATCC 27853). Each strain was incubated in a thermostat at 37 °C *o.n*. Subsequently, some colonies were diluted in Phosphate-Buffered Saline (PBS) solution (VWR International S.r.l., Radnor, PA, USA) and measured with a DEN-1B densitometer (Biosan, Riga, Latvia) up to a turbidity of 0.5 McFarland.

Scalar 1:10 dilutions of the inoculum were made to obtain a solution with a concentration of about 1.5 × 10^6^ CFU/mL. Then, 300 μL of the above dilution was placed inside each well and exposed to radiation under room temperature (≈22 °C) and humidity of ≈36%. The 300 µL volume in the well results in a liquid column height of approximately 3–4 mm. Given the high penetration of 405 nm radiation in clear aqueous buffers, the dose delivered is considered uniform throughout the volume. At the end of the exposure, 1:10 scalar dilutions of the exposed samples were made, and an aliquot of 100 μL (approximately 1.5 × 10^3^ CFU) was dispersed with the use of a sterile spatula (VWR International S.r.l., Radnor, PA, USA) until completely absorbed on plates with solid Plate Count Agar (PCA) medium (VWR International, Radnor, PA, USA). To isolate the germicidal contribution of the 405 nm radiation, a parallel control strategy was implemented. For every exposure interval, a ‘positive control’ was maintained under identical environmental conditions (temperature, humidity, and chemical stressor concentration). Logarithmic abatement was calculated using the formula: log_10_ (CFU control/CFU exposed). This method accounts for any potential spontaneous bacterial reduction unrelated to light exposure, ensuring that the reported results strictly represent the radiation-induced inactivation. At the end of exposure, all samples and their respective controls were incubated at 37 °C for 48 hours (h). CFU counts were performed using a SCAN 100 manual colony counter (Interscience, Saint Nom la Bretèche, France).

Bacterial strains were exposed to increasing doses of light energy to make statistical curves depicting the relationship between the administered energy dose and the abatement level. The plate was placed on top of the black PLA support at about 10 cm from the light source radiating from below. Doses (*D*) of energy administered were calculated using the formula *D = E_t_* × *I_R_*, where *E_t_* stands for “exposure time” and *I_R_* stands for “irradiance”, expressed in J/cm^2^. Administered energy was chosen according to the bacterial strain to be tested (and consequently the exposure times), based on literature references [15,20,21,22] (Table 1).

### 2.3. Sub-Lethal Stress Condition

Under stress conditions, microorganisms remain viable but employ adaptive responses to new stimuli that generally lead to altered growth parameters, with sub-lethal injury. In our study, we evaluated the effects of osmotic, thermal and oxidative stress conditions on bacterial cultures in the presence and absence of light radiation at 405 nm.

#### 2.3.1. Osmotic Stress

Bacterial cultures at a concentration of 1.5 × 10^6^ CFU/mL, exposed and unexposed to *near*-UVA light, were suspended in 15% P/V (*w*/*v*) sodium chloride (NaCI) salt solution (ITWreagents S. r. 1., Monza, Italy). Multiwell plates not exposed to the light source, which were used as controls, were maintained under the same temperature (≈22 °C) and humidity (≈36%) conditions as the exposed plates (plate setup procedures and exposure doses can be found in Section 2.3 and Table 1). The results were compared with the inactivation curves of microorganisms exposed to light at room temperature (≈22 °C) and with the inactivation curves of control samples.

#### 2.3.2. Thermal Stress

Bacterial cultures at a concentration of 1.5 × 10^6^ CFU/mL in PBS were maintained at 4 °C (in a refrigerator) and 45 °C (in an incubator). For heat stress, suspensions were prepared as described previously (Section 2.3) and exposed to light at 405 nm at the doses in Table 1. The results were compared with the inactivation curves of microorganisms exposed to light at room temperature (≈22 °C) and the inactivation curves of control samples.

#### 2.3.3. Oxidative Stress

For oxidative stress, a 9 mM solution in PBS of the exogenous porphyrin TMPyP4-4CI (5,10,15,20-(tetra-N-methyl-4-pyridyl)porphyrin tetrachloride) (PorphyChem SAS, Longvic, France) was prepared. This molecule belongs to the class of cationic tetra-substituted porphyrins. Its mechanism of action is mediated by a Type II photochemical pathway: upon excitation by 405 nm light (Soret band), it transfers energy to molecular oxygen, generating highly reactive singlet oxygen (^1^O_2_). The cationic nature of the molecule ensures strong electrostatic affinity for the negatively charged bacterial cell walls, particularly lipopolysaccharides in Gram-negative and teichoic acids in Gram-positive bacteria, leading to localized oxidative damage [23,24]. 

The porphyrin was solubilized in 1X PBS to maintain a stable pH of 7.4. TMPyP4 demonstrates high solubility in aqueous media at this pH, attributable to its four positive charges. The final pH of the bacterial suspension was maintained between 7.2 and 7.4, thereby ensuring that the porphyrin stayed in its active, monomeric form and that aggregation was prevented, thus avoiding any potential diminution of the photodynamic effect.

Bacterial suspensions at a concentration of 1.5 × 10^6^ CFU/mL were incubated for 10 min with the porphyrin at a final concentration of 9 nM. The multiwell plate setup with the bacterial suspension and porphyrin was exposed to near-UVA light as described in Section 2.3. The unexposed control plate was kept in the dark and under the same environmental conditions as the light-exposed plate. Results were compared with the inactivation curves of the microorganisms exposed to light at room temperature (≈22 °C) and the inactivation curves of the control samples.

### 2.4. Statistical Analysis

For statistical analysis, the data obtained from the experiments were entered into a database, with the types of treatment tested divided into classes. Then, the classes were merged by type into 3 groups of experiments: (i) near-UVA light only, (ii) near-UVA light and sub-lethal stress, and (iii) sub-lethal stress only.

Microsoft Excel (2016 version) was used to collect data and organize the database. The culling results were calculated as the logarithm of the ratio of the CFUs of the samples to those of the positive controls compared to the individual energy doses. Statistical analysis was performed using the nonparametric Kruskal–Wallis test with the STATA 17 software (StataCorp LLC, College Station, TX, USA) by checking the equality of the medians ($\tilde{x}$) of different groups and comparing the antimicrobial effect of near-UVA radiation in the presence and absence of sub-lethal conditions for individual bacterial species.

All the data collected during the study were entered into a database and included the following variables: Date of test, Petri dish ID, CFUs/mL, CFUs/m^3^, microorganism species and inoculum concentrations. Descriptive statistics of the empirical data were calculated as mean of log_10_ CFU reduction together with 95% confidence intervals to evaluate the reliability of the measurements.

## 3. Results

Significant differences in reduction were observed between the different bacterial strains in the n-UVA light-only tests. For *E. coli* and *E. faecalis*, an inactivation of 0.68 log_10_, with an Interquartile Range (IQR 25–75°) of 0.62–0.8 and 0.81 (0.62–1.09), respectively, was achieved at the maximum energy doses (360 and 250 J/cm^2^), corresponding to 83% and 85% reductions in CFU compared to positive controls.

For *P. aeruginosa*, the median inactivation at the highest dose (250 J/cm^2^) was 2.51 log_10_ (IQR 1.71–2.95), corresponding to a 99.6% reduction. Finally, for *S. aureus*, a median of 0.5 log_10_ (IQR 0.41–0.52) was achieved, corresponding to an in vitro microbial load reduction of approximately 66%.

The efficacy of the treatment was significantly improved by exposing osmotically stressed bacterial suspensions (NaCl 15%) to light irradiation: for *E. coli* and *E. faecalis* at the highest dose, the median reduction increased to 2.76 log_10_ (IQR 2.42–2.84) and 5.46 log_10_ (IQR 5.43–5.47), respectively, achieving a 98.7% and over 99.999% reduction in microbial concentration.

For *P. aeruginosa* and *S. aureus*, treatment resulted in 5.31 (IQR 5.23–5.42) and 1.50 log_10_ (IQR 1.03–1.89) inactivation, respectively, compared to untreated controls. Treatment with a combination of light and heat stress, at a temperature of 4 °C, resulted in a 1.11 log_10_ (IQR 1.05–1.23) reduction in *E. coli*. Compared to near-UVA light alone and in combination with osmotic stress, this result is intermediate. For *E. faecalis*, under the same conditions, the reduction was 0.24 log_10_ (IQR 0.18–0.27). Higher reductions were observed for *P. aeruginosa* and *S. aureus*: 2.7 (IQR 2.47–3.05) and 5.50 log_10_ (IQR 5.48–5.52), respectively. The efficacy of near-UVA irradiation in combination with heat at 45 °C was higher for all strains compared to the previous condition. For *E. coli*, we obtained a median inactivation of 2.09 log_10_ (IQR 1.8–2.42), while for *E. faecalis* the median was 0.58 log_10_ (IQR 0.48–0.66), confirming it as the most resistant of the bacterial strains tested. Elevated temperature was particularly effective against *P. aeruginosa* and *S. aureus*, with median reductions of 5.34 log_10_ (IQR 5.30–5.35) and 5.37 log_10_ (IQR 5.30–5.41) at doses of 100 and 126 J/cm^2^, respectively, corresponding to reductions of over 99.999%.

Tests performed by exposing the strains to light radiation under conditions of oxidative stress, induced by the addition of the exogenous porphyrin, gave different results depending on the species. In fact, the median reduction for *P. aeruginosa* at the lowest dose (50 J/cm^2^) was 5.41 log_10_ (IQR 5.37–5.45), while the median reductions for *E. coli* and *E. faecalis* were 0.42 log_10_ (IQR 0.28–0.48) and 0.37 log_10_ (IQR 0.20–0.55), respectively. A median reduction of 5.45 log_10_ (IQR 5.43–5.49) was also observed for S. aureus at an intermediate dose of 150 J/cm^2^.

The non-parametric Kruskal–Wallis test was used to assess the significance of the medians between the different treatment classes and between the different experimental groups. For each microbe, all comparisons made (between classes and groups) were statistically significant (*p* = 0.001).

**Figure 4:**
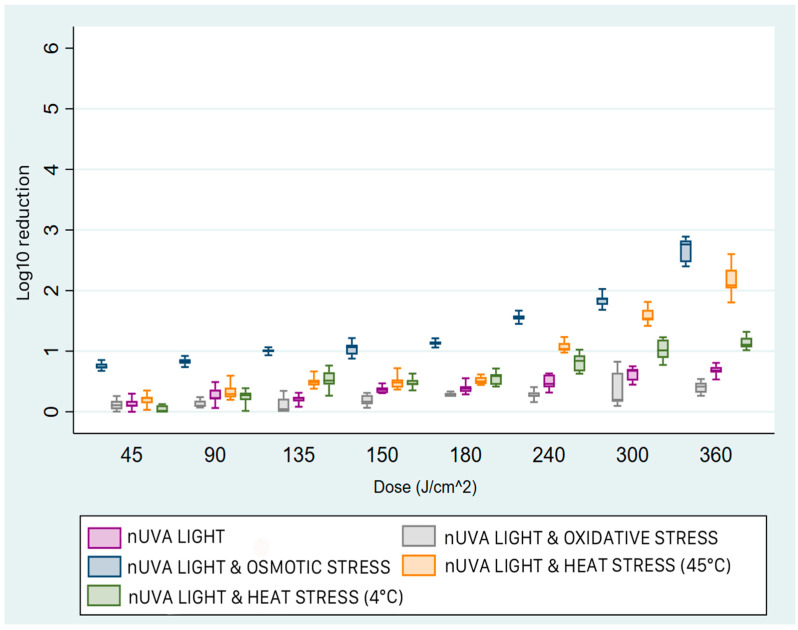
Box plots of the reduction achieved with near-UVA light and sub-lethal stress on *E. coli*. Comparing the different treatments, exposure to near-UVA light with osmotic stress (blue box) was the most effective; thermal stress at 4 °C and 45 °C (green and orange boxes, respectively) produced intermediate values, and the treatment with light and oxidative stress (grey box) was the least effective. The box represents the interquartile range (IQR) of bacterial reduction. Within the two quartiles, the median value of the reduction is shown. The upper whisker ends at the maximum value, and the lower whisker ends at the minimum value achieved.

For *E. coli*, the most effective treatment was exposure to light radiation under osmotic stress, followed by thermal stress at 45 °C and 4 °C, and finally oxidative stress (Figure 4). By increasing or decreasing the temperature, the median concentration reduction remained almost constant up to a dose of 180 J/cm^2^ ($\tilde{x}$ = 0.5 log_10_, IQR 0.4–0.57). As the energy dose increased, the reduction gradually increased: to a median of 2.08 log_10_ (1QR 1.98–2.34) and slightly more than 1.0 log_10_ (IQR 1.05–2.34) when the samples were treated at 4 °C. Treatment with exogenous porphyrin resulted in lower abatement than the other conditions at all doses. In fact, the median reduction was 0.41 log_10_ (IQR 0.29–0.48), achieved only at the highest energy dose administered (360 J/cm^2^).

**Figure 5:**
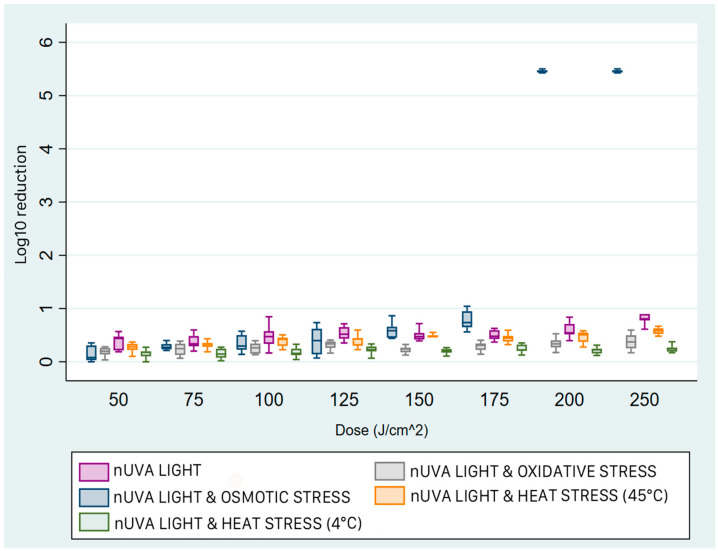
Box plots of the reduction achieved with near-UVA light and sub-lethal stress on *E. faecalis*. For the different treatments, near-UVA light with osmotic stress (blue box) was the most effective starting at a dose of 150 J/cm^2^, but before reaching peak knockdown at a dose of 200 J/cm^2^, the trend of logarithmic reduction increased with the dose administered. In contrast, treatment with light and heat stress at 4 °C (green box) was the least effective. The box represents the interquartile range (IQR) of bacterial reduction. Within the two quartiles, the median value of the reduction is shown. The upper whisker ends at the maximum value, and the lower whisker ends at the minimum value achieved.

*E. faecalis* was the most resistant strain to light irradiation (Figure 5). In the presence and absence of stress conditions, the inactivation values were less than 1 log_10_, except for osmotic stress. In fact, no other combination of light and stress resulted in a greater reduction in concentration than radiation alone. Under conditions of increased salt concentration, significant reductions were achieved with doses of 200 J/cm^2^ and 250 J/cm^2^ ($\tilde{x}$ = 5.46 log_10_; IQR 5.43–5.48).

**Figure 6:**
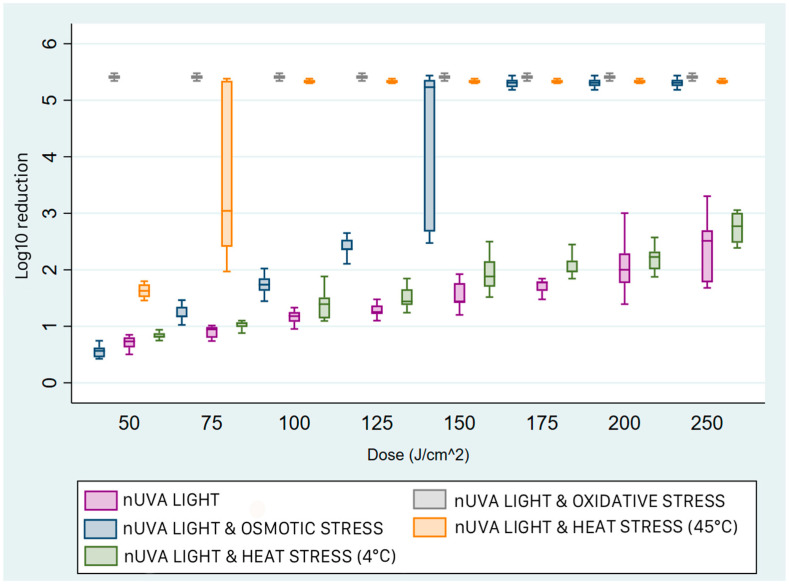
Box plots of the reduction achieved with near-UVA light and sub-lethal stress on *P. aeruginosa*. Comparing the different treatments, near-UVA light with oxidative stress (grey box) was the most effective at the lowest dose (50 J/cm^2^). The treatments with light and heat stress at 45 °C (orange box) and with oxidative stress (blue box) produced a reduction equal to that of oxidative stress at the highest doses. The reductions with light and heat stress at 4 °C (green box) and with light alone (purple box) were about 2.5 log_10_ lower. The box represents the interquartile range (IQR) of bacterial reduction. Within the two quartiles, the median value of the reduction is shown. The upper whisker ends at the maximum value, and the lower whisker ends at the minimum value achieved.

The results obtained with *P. aeruginosa* show a marked sensitivity of the strain to the addition of exogenous porphyrin in samples exposed to light even at the lowest dose (Figure 6).

Similar reductions were obtained with temperature at 45 °C, where a median reduction of 5.34 log_10_ (IQR 5.30–5.35) was achieved at 100 J/cm^2^. For osmotic stress, an upward trend proportional to the energy dose administered was observed, reaching a maximum peak reduction at 175 J/cm^2^. At 4 °C, the median reduction was 2.7 log_10_ (IQR 2.47–3), slightly higher than that obtained with light radiation alone ($\tilde{x}$ = 2.51; IQR 1.75–2.8).

**Figure 7:**
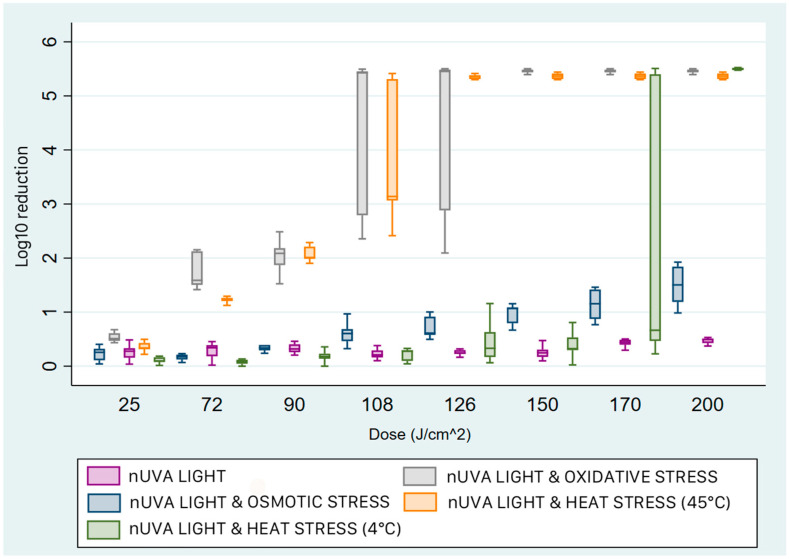
Box plots of the reduction achieved with near-UVA light and sub-lethal stress on *S. aureus*. Near-UVA light with oxidative stress (grey box) reduced the microbial concentration at the lowest doses more than the other treatments. The combination of light and heat stress at 45 °C (orange box) and 4 °C (green box) only achieved abatement levels of more than 5 log_10_ (as did oxidative stress) above a dose of 126 J/cm^2^. The combination of near-UVA radiation and osmotic stress (blue box) proved to be less effective than the previous treatments, while light radiation alone (purple box) was the least effective. The box represents the interquartile range (IQR) of bacterial reduction. Within the two quartiles, the median value of the reduction is shown. The upper whisker ends at the maximum value, and the lower whisker ends at the minimum value achieved.

For *S. aureus*, oxidative stress and heat stress at 45 °C in combination with light were the most effective treatments. In fact, for both we obtained a reduction of 5.36 log_10_ (IQR 5.30–5.41 for oxidative; 5.30–5.39 for 45 °C) at the dose of 126 J/cm^2^. The combination of light and heat stress at 4 °C was more effective than osmotic stress only at the dose of 170 J/cm^2^ ($\tilde{x}$ = 0.66; IQR 0.36–5.45). Exposure to light radiation in combination with the addition of 15% NaCl produced a median of 1.5 log_10_ (IQR 1.10–1.86) at the highest energy dose. The level of reduction in microbial concentration increased with increasing energy dose. Exposure to near-UVA radiation alone was significantly less effective for this strain than combinations with sub-lethal stresses ($\tilde{x}$ = 0.49 log_10_; IQR 0.41–0.51) (Figure 7).

## 4. Discussion

The results obtained from the exposure of the bacterial strains analysed to 405 nm near-UVA radiation show lower levels of reduction than those reported in the literature.

For *P. aeruginosa*, the maximum inhibition value obtained was 2.51 log_10_ at 250 J/cm^2^, whereas higher inhibition values were found in the literature at lower energy doses [20,22].

For *E. coli*, *E. faecalis* and *S. aureus*, the reductions achieved in the tests were all less than 1 log_10_, about 2–4 log_10_ lower than in reference studies [15,25,26].

For *S. aureus*, the reduction at a dose of 200 J/cm^2^ ($\tilde{x}$ = 0.51 log_10_) is about half the reduction achieved in another study at a dose of 108 J/cm^2^ [21].

The characterisation of the emission spectrum of the light source could partly explain the discrepancy between the values obtained and those expected. Indeed, while in our experiments the peak irradiance of LEDs is centred around the frequency of 405 nm with a bandwidth of ±5 nm, in the cited works this range is wider (±10 to ±25 nm) [15,21,27,28]. At higher energy frequencies, more endogenous photosensitive molecules are excited, and thus the degree of microbial reduction following radiation exposure is greater.

Different stresses (osmotic, thermal and oxidative) have detrimental but not lethal effects on cells in the absence of light. In fact, bacteria activate metabolic and structural adaptive response mechanisms [29]. The increased levels of intracellular ROS produced by radiation at 405 nm, when combined with the effects of stress, resulted in overall higher levels of reduction than when light radiation was used alone.

Under osmotic stress, *E. coli* increases the expression of OmpX, a protein that regulates outer membrane components such as porins and efflux pumps [30], reducing water loss and cell lysis. This also limits the scavenging of ROS generated by 405 nm radiation. The addition of 15% NaCl resulted in a reduction of over 2.5 log_10_ for all bacteria except *S. aureus* ($\tilde{x}$ = 1.5 log_10_). The halotolerant nature of *Staphylococcus*, which allows cation binding to the membrane and restricts Na^+^ ion entry, may explain its ability to survive higher salt concentrations than ambient conditions [31].

Heat stress (at 4 °C and 45 °C) combined with near-UVA resulted in higher knockdowns than exposure at ambient conditions, except for *E. faecalis*. Exposure to low or high temperatures induces structural changes in bacteria, including loss of membrane fluidity, protein unfolding, accumulation of ribosomal subunits and negative DNA supercoiling. These changes activate the cold shock response (CSR) and heat shock response (HSR) mechanisms [32]. CSR involves the CspA family of proteins, found in *E. coli* and most bacteria, which regulate functions such as increasing unsaturated fatty acids in membranes to restore fluidity [33]. In *S. aureus*, CspA is involved in the response to antimicrobial peptides and the regulation of stress-related proteins [34]. HSR is activated by thermoregulatory mechanisms involving different biomolecules such as lipids, proteins and nucleic acids [35]. The correlation between membrane changes and cellular responses suggests that oxidative damage to membrane lipids by ROS after near-UVA exposure leads to cell death. Temperature reduction induces protein synthesis, including riboflavins [36], which are targeted by UVA and near-UVA radiation, possibly explaining the greater microbial reduction compared to ambient treatment [37].

When bacterial suspensions are incubated with an exogenous porphyrin, it can: (i) settle outside the cell and generate ROS in solution that damage cell membranes, (ii) bind to cell membranes and modify them, or (iii) penetrate inside the cells by binding to different substrates [38]. In our experiments, the reduction in microbial concentration achieved by the addition of exogenous porphyrin at a concentration of 9 nM differed significantly depending on the species tested. In fact, although we obtained reductions of more than 5 log_10_ for *P. aeruginosa* and *S. aureus*, for *E. coli* and *E. faecalis* the reductions were significantly lower ($\tilde{x}$ = 0.42 and 0.47 log_10_), even lower than those obtained by exposing the bacterial suspensions to light radiation alone. Even in the literature, the reduction of *E. faecalis* by near-UVA radiation in the presence of various exogenous photosensitizers at micromolar concentrations (about 1000 times higher than the concentration used in this work) does not exceed 1 log_10_ [34]. This variability is unlikely to be due to structural differences between the species, as *E. faecalis* and *E. coli* are Gram-positive and Gram-negative, respectively. A possible explanation lies in the different ways in which the species internalise the endogenous photosensitive molecule. Indeed, *E. faecalis* has numerous resistance genes located on mobile elements such as plasmids and transposons encoding channel and transport proteins [39]. Thus, the reduction values obtained with *E. coli* and *E. faecalis* could be due to their greater ability to synthesise efflux pumps than the other two species tested [40,41]. In conclusion, for the exposure of the bacterial suspensions to near-UVA radiation, the best reduction was obtained with *P. aeruginosa*.

Combining light at 405 nm with the different types of stress resulted in higher overall reductions, particularly in the case of oxidative stress. For all species (except *E. faecalis*), varying the temperature to 4 and 45 °C resulted in higher reductions on average. In the case of oxidative stress induced by exogenous porphyrin, only *P. aeruginosa* and *S. aureus* showed a significant susceptibility to treatment. The experiments carried out show that the use of near-UVA light has considerable potential in the field of environmental disinfection, especially if appropriate energy doses, preferably based on the results obtained, are used in combination with osmotic and thermal stress at 45 °C. Although the presence of near-UVA light generally demonstrates its supporting role in environmental disinfection [42] in synergy with the “classical” chemical solvent approach, the exclusive use of near-UVA light still needs to be further investigated. Characterisation of the cellular response using molecular techniques could facilitate the identification of genetic factors that could lead to efficient treatment with the combination of near-UV light and exogenous porphyrins across different bacterial species.

The results of this investigation confirm the efficacy of this wavelength for optical disinfection. By integrating near-UVA radiation with osmotic and thermal stressors, we observed an inhibitory effect surpassing the combined impact of each factor individually, indicating a synergistic disruption of bacterial homeostasis. This approach is especially pertinent to environmental sustainability: porphyrins are organic, biodegradable photosensitizers that do not present a risk of bioaccumulation, unlike conventional halogenated disinfectants. Furthermore, the 405 nm wavelength, commonly referred to as high-intensity narrow-spectrum (HINS) light, functions outside the UVC spectrum, providing a safer option for disinfecting occupied areas and delicate materials.

### Study Limitations

This study, although thorough in its investigation of the effects of near-UVA light on bacterial strains, has some limitations. Firstly, the narrow wavelength range of the experimental setup (405 nm with a bandwidth of ±5 nm) differs from other studies, which may contribute to the lower microbial reduction levels observed. However, this precise control over the wavelength ensures consistency and reproducibility of results within the defined parameters.

Secondly, the study focused on four specific bacterial strains, which limits the generalisability of the results to other strains. Nevertheless, the selected strains represent common pathogens and provide relevant insights into the efficacy of near-UVA light against these bacteria.

While useful for understanding bacterial responses, the sub-lethal stress conditions used in the experiments may not fully replicate the complex environmental stresses encountered in real-world settings. Nevertheless, these controlled conditions allow a clear assessment of the effects of near-UVA light under specific stressors and provide valuable preliminary data.

Finally, the study did not investigate the long-term effects of near-UVA exposure or potential adaptive resistance in bacteria, which could affect the sustainability of this disinfection method. Future research could address these aspects to confirm the practicality and efficacy of near-UVA light for long-term environmental disinfection. Despite these limitations, the study provides important insights and a basis for further exploration of near-UVA light as a disinfection tool.

## Figures and Tables

**Table 1:** Doses of light energy administered in different samples for different bacterial strains.

Strain	Doses per Sample (J/cm^2^)							
	1	2	3	4	5	6	7	8
*S. aureus*	25	72	90	108	126	150	170	200
*E. faecalis*	50	75	100	125	150	175	200	250
*P. aeruginosa*	50	75	100	125	150	175	200	250
*E. coli*	45	90	135	150	180	240	300	360

## Data Availability

The datasets used and/or analysed during the current study are available from the corresponding author upon reasonable request.

## Abbreviations

The following abbreviations are used in this manuscript:

HCAIs	Healthcare-Associated Infections
HAIs	Healthcare-Associated Infections
ETO	Ethylene oxide
ROS	Reactive Oxygen Species
LED	Light Emitting Diodes
PLA	Polylactic acid
PBS	Phosphate-Buffered Saline
PCA	Plate Count Agar
IQR	Interquartile Range
CSR	Cold Shock Response
HSR	Heat Shock Response
